# Retrofit-induced changes in the radiated noise and monopole source levels of container ships

**DOI:** 10.1371/journal.pone.0282677

**Published:** 2023-03-16

**Authors:** Vanessa M. ZoBell, Martin Gassmann, Lee B. Kindberg, Sean M. Wiggins, John A. Hildebrand, Kaitlin E. Frasier

**Affiliations:** 1 Scripps Institution of Oceanography, University of California San Diego, La Jolla, California, United States of America; 2 Maersk North America, Charlotte, North Carolina, United States of America; Wildlife Conservation Society Canada, CANADA

## Abstract

The container shipping line Maersk undertook a Radical Retrofit to improve the energy efficiency of twelve sister container ships. Noise reduction, identified as a potential added benefit of the retrofitting effort, was investigated in this study. A passive acoustic recording dataset from the Santa Barbara Channel off Southern California was used to compile over 100 opportunistic vessel transits of the twelve G-Class container ships, pre- and post-retrofit. Post-retrofit, the G-Class vessels’ capacity was increased from ~9,000 twenty-foot equivalent units (TEUs) to ~11,000 TEUs, which required a draft increase of the vessel by 1.5 m on average. The increased vessel draft resulted in higher radiated noise levels (<2 dB) in the mid- and high-frequency bands. Accounting for the Lloyd’s mirror (dipole source) effect, the monopole source levels of the post-retrofit ships were found to be significantly lower (>5 dB) than the pre-retrofit ships in the low-frequency band and the reduction was greatest at low speed. Although multiple design changes occurred during retrofitting, the reduction in the low-frequency band most likely results from a reduction in cavitation due to changes in propeller and bow design.

## Introduction

Ambient noise levels in the ocean have risen over the past five decades due, in part, to a tripling in the number of vessels in the world’s merchant fleet [1–4]. With the goal of mitigating noise impacts on marine organisms, a number of source-centric efforts to reduce underwater noise emitted from commercial ships have been implemented on regional scales through vessel speed reduction programs near ports [5, 6], as vessel speed and noise levels are positively correlated [7–9]. To reduce underwater noise generated by anthropogenic sources on a larger spatial scale and across national boundaries, the potential for individual vessel noise reductions are being discussed by the International Maritime Organization (IMO), International Whaling Commission (IWC), and the International Union for Conservation of Nature (IUCN) [10]. Retrofitting commercial ships with specific propeller and hull design modifications has been identified by the IMO as a potential method to reduce underwater noise from commercial ships on an international scale [11, 12].

**Fig 1:**
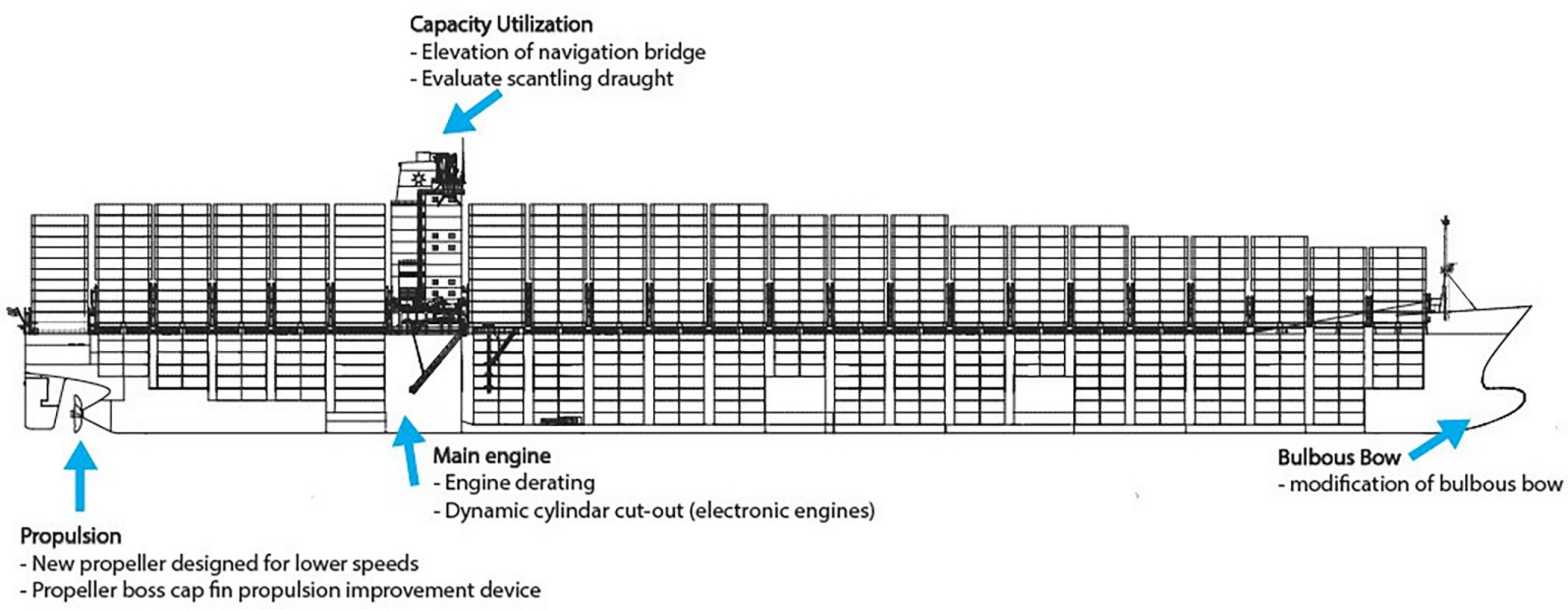
G-Class vessel post-retrofit. G-Class vessel post-retrofit, modified from the original blueprint provided by Maersk. Changes from pre- to post-retrofitting are identified with arrows.

Maersk is the world’s largest container ship operator in both fleet size and cargo capacity [13, 14]. Maersk has grown to a fleet of over 700 container ships, serving 59,000 customers around the world, with access to 343 port terminals [13, 14]. Maersk has completed a $1 billion, 5-year “Radical Retrofit” initiative focused on improving energy efficiency and fuel consumption to reduce emissions. During this effort, twelve G-Class sister ships, designed for transport of containers, were retrofitted from 2015 through 2018. The Radical Retrofit program included redesigning the bulbous bow to reduce drag and derating the engine to improve vessel efficiency at slower speeds. Additionally, the propeller blade number was reduced from 6 to 4, engine rpm was increased by 10%, propeller diameter increased from 9 to 9.3 m, area and thickness of the propeller blades was reduced, and propeller boss cap fins were installed to reduce cavitation. Each vessel’s container capacity was increased from ~9,000 twenty-foot equivalent units (TEUs) to ~11,000 TEUs, which also increased the vessel draft (Fig 1).

Reduction of underwater noise was identified as a potential added benefit of emission reductions following the Radical Retrofit program [15]. To compare the underwater noise of these container ships pre- and post-retrofit, long-term acoustic recording datasets in heavily trafficked shipping lanes were used. Over the past ten years, the Scripps Institution of Oceanography opportunistically recorded over 100 transits of the twelve G-Class Maersk vessels pre- and post-retrofitting as they transit through the Santa Barbara Channel (SBC) to and from the Ports of Los Angeles and Long Beach. In this study, the changes in radiated noise levels and monopole source levels after retrofitting are investigated.

## Methods

### Ship passages

Automatic identification system (AIS) receiver stations were operated in the Santa Barbara Channel to determine ship locations in this study (Table 1). The IMO number of each G-class vessel was used to search these AIS databases for passages that were within 5 km of an acoustic recording device used in this study. When a passage from one of the twelve G-Class vessels was identified, corresponding information such as Speed Over Ground (SOG), Course Over Ground, and position (longitude and latitude) were decoded from the AIS messages. Speed Over Ground was converted to Speed Through Water (STW) by accounting for speed and direction of surface currents from High-Frequency (HF) Radar data from the Southern California Coastal Ocean Observing System [16].

**Table 1:** Automated identification system data sources.

AIS Station	Latitude	Longitude	Date Range
UC Santa Barbara	34.408° N	119.878° W	June 2007—May 2013
Santa Cruz Island	33.994° N	119.632° W	March 10—October 2017
Coal Oil Point	34.411° N	119.877° W	September 2013—June 2018
Santa Ynez Peak	34.029° N	119.784° W	August 2016—present

Automated Identification System (AIS) station locations and dates for records.

### Acoustic recordings

**Fig 2:**
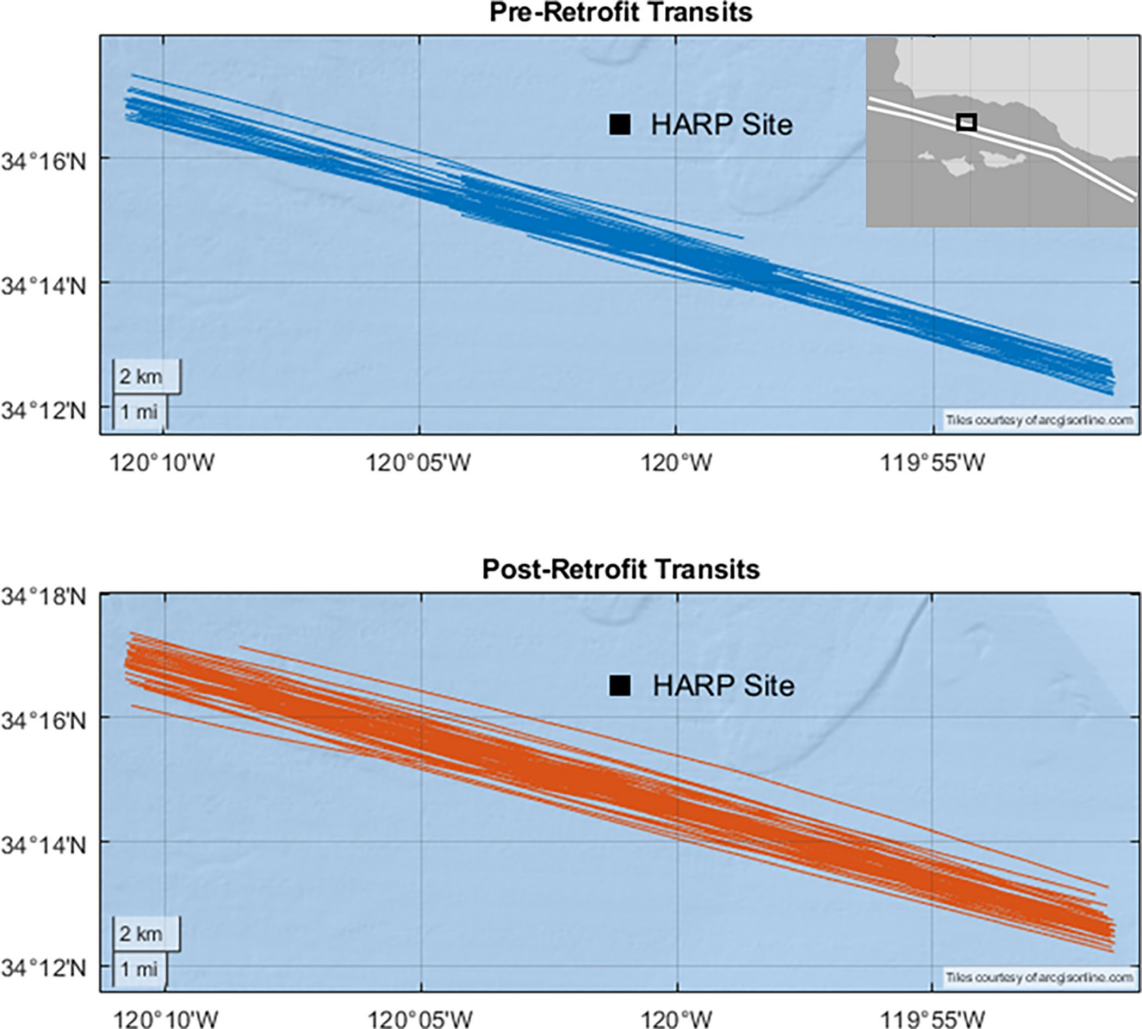
Study site in the Santa Barbara Channel. Top: Pre-retrofit transits are shown in blue. Bottom: Post-retrofit transits are shown in red. The location of the High-frequency Acoustic Recording Package (HARP) is labeled with a square. The inset map in the upper right corner shows the north-south Traffic Separation Scheme as white lines.

A High-frequency Acoustic Recording Package (HARP) was maintained in the Santa Barbara Channel (Site B; 34.270° N, 120.030° W) at ~580 m depth, ~3 km north of the northbound shipping lane from February 2008 to November 2018 (Fig 2) [17]. The HARP was equipped with a single, omni-directional hydrophone that was suspended 10 m above the seafloor. HARP hydrophone electronics were calibrated at Scripps Institution of Oceanography and representative hydrophones were calibrated at the U.S. Navy’s Transducer Evaluation Center facility in San Diego, California. Recordings were collected at a sampling rate of 200 kHz. Acoustic data were decimated by a factor of 20 to reduce computational requirements. The data were low-pass filtered with an 8th order Chebyshev Type I IIR filter during decimation to prevent aliasing and then resampled at 10 kHz. Identified G-Class vessel transits from the AIS data were paired with corresponding acoustic recordings and extracted for manual review by a trained analyst (VMZ). Transits contaminated with low-frequency hydrophone cable strumming, electronic noise from the instrument, marine mammal vocalizations, or ship noise from another vessel were discarded. Frequencies associated with electronic noise were excluded from the spectra and broadband calculations.

### Vessel noise metrics

At speeds corresponding to commercial operation of the ships (e.g., 5–10 m/s), the most energetic source of vessel noise is propeller cavitation [4], that is, the creation of a water cavity by reduction in local pressure due to motions of the propeller. Because cavitation involves a water volume change, it is a monopole sound source, but since propellers are operated near the surface of the ocean (at least for surface vessels) reflections from the sea surface result in an image sound source with reversed polarity, called the Lloyd’s mirror effect, effectively creating a dipole sound source. At low-frequency (i.e. when the distance between the monopole and sea surface is small by comparison to the acoustic wavelength), a dipole sound source has directionality and the strength of radiated energy varies with angle as cos(theta) where theta is the normal angle with respect to the sea surface. Vessel radiated noise levels (L_RN_) are calculated in a manner that does not take into account the source depth, nor the dipole nature of the source. Instead, an explicit account of the dipole source is calculated by the monopole source level, which is a better metric for the energy due to cavitation or other near-surface sources estimated 1 meter away from the source [18].

Radiated noise levels (L_RN_) were calculated and monopole source levels (L_S_) obtained by correcting for the effect of Lloyd’s mirror using the approach of Gassmann et al. (2017) [18]. ASA/ANSI (2009) and ISO (2019) standard measurement methods were adhered to, with the exception of observation angles, which were not controlled for during these opportunistic recordings [19, 20]. Measurements of the twelve sister ships were conducted at ranges varying from 1.7–4.9 km; therefore, the vertical and horizontal observation angles deviated from the ASA/ANSI (2009) requirements [19]. To estimate monopole source levels at 1 m range, frequency-dependent sound pressure levels (L_P_) were measured at and near the closest point of approach (d_CPA_), and a frequency-dependent propagation loss model (N_PL_) with a unique source depth (d_S_) was applied with the following equation:

$$L_{S}=L_{P}+N_{PL}$$ (1)

### Sound Pressure Level (L_P_)

Each G-Class vessel recording was divided into non-overlapping segments with a duration of 1 s, and a 10,000-point Fast Fourier Transform (FFT) applied to each 1 s segment to provide a frequency bin spacing of 1 Hz. Over the duration of the transit, the mean sound pressure level was averaged over 5 s segments every 3 s to smooth the time-frequency distribution. The resulting sound pressure levels (L_P_) were reported in decibels (dB) with a reference pressure of 1 μPa^2^. Ambient noise levels in the absence of ships were calculated for each deployment as the 10^th^ percentile of daily ambient noise over the course of each deployment and were compared to L_P_ values to compute signal to noise ratios for each transit.


$$L_{P}=10log10\left(\frac{p^{2}}{p_{0}}\right)\;dB$$ (2)

### Propagation loss

A variety of propagation loss models have been applied to vessels transiting in the Santa Barbara Channel [6, 7, 18, 21]. To estimate L_RN_, a spherical spreading propagation loss model (N_SS_) was calculated with the following equation:

$$N_{SS}=20log10\left(\frac{R}{r_{0}}\right)\;dB$$ (3)

where R is the distance from the dipole source to the receiver and r_0_ is the reference distance (1 m).

Additionally, a propagation loss model that corrects for the Lloyd’s mirror effect (N_PL_) was applied to account for sea surface image source interference, in compliance with ISO (2019) [20]. The N_PL_ model ignores sound refraction in the water column and reflections from the seafloor and solely accounts for reflections from the sea surface [18]. The propagation loss of a sound source near the surface in deep water considering the Lloyd’s mirror effect is given by:

$$N_{PL}=-20log10\left(r_{0}|\frac{e^{-jkr_{1}}}{r_{1}}-\frac{e^{-jkr_{2}}}{r_{2}}|\right)\;dB$$ (4)

where r_1_ is the distance from the source to the receiver, r_2_ is the distance from the image source to the receiver, and k is the wave number in rad/m. Harmonic mean sound speeds were calculated from depth, temperature, and salinity data obtained from the California Cooperative Oceanic Fisheries Investigations (line 81.8, station 46.9) and California Underwater Glider Network using the nine term equation from Mackenzie (1981) [22–24].

A modification of the Lloyd’s mirror model was applied to remove mismatched interference lobes identified with ship noise measurements in compliance with ANSI/ASA (2009) and ISO (2019) [18–20]. The modification involves using the Lloyd’s mirror model from 5 Hz up to the lowest frequency at which the Lloyd’s mirror model (Eq 4) and the spherical spreading model (Eq 3) intersect, while at the higher frequencies, the spherical spreading model was used [18].

### Source depth (d_S_)

**Fig 3:**
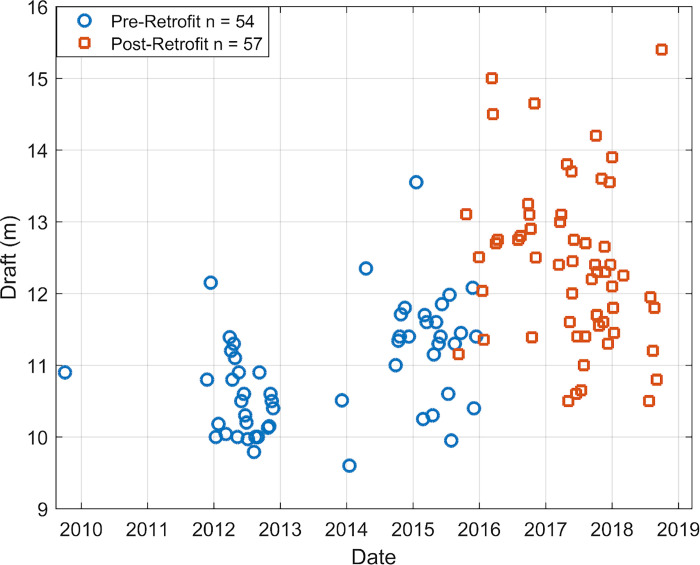
G-Class Maersk vessel draft measurements. G-Class Maersk vessel draft measurements pre-retrofit (blue circles) and post-retrofit (red squares), provided by vessel Chief Engineers. Pre-retrofit draft measurements were shallower on average than post-retrofit draft measurements.

The spherical spreading propagation loss model used to compute radiated noise level does not require a source depth (d_S_). Multiple types of cavitation can occur at various locations around the propeller, including tip, blade, and hub vortex, making any method of source depth calculation subject to some error. To correct for changes in d_S_ when calculating monopole source level, ISO (2019) recommends a d_S_ equal to 70% of the vessel draft [20]. Approximating the source depth by a percentage of the vessel draft does not take into account the propeller diameter, an important factor in d_S,_ since cavitation is most prominent at the propeller tip at the top of its rotation [25]. Propeller diameter measurements are challenging to obtain, and are not reported in Lloyd’s Register of Ships, or any publicly available datasets. By communicating with the vessel designers, we obtained the propeller diameters of each G-Class vessel pre- and post- retrofitting (9 m and 9.3 m, respectively); therefore, we used a d_S_ equal to 85% of the propeller diameter subtracted from the ship draft [25]. Draft measurements were obtained from the Chief Engineers (CEs) of each ship and compared to those reported in the AIS data. The AIS reported draft measurements were up to 3 m different from the draft measurements obtained from the CEs of the ships; therefore, the CEs’ measurements were used as these were considered more reliable. The draft measurements from the CEs for each transit are shown in Fig 3.

### Radiated Noise Level (L_RN_) and Source Level (L_S_)

Sound pressure levels (L_P_) for each G-Class vessel transit were obtained by averaging over the time it took the ship to travel +/- 30 degrees with respect to the d_CPA_, adhering to ANSI/ASA, 2009. Radiated noise level (L_RN_) was determined by applying spherical spreading loss (N_SS_) to L_P_, and monopole source level (L_S_) was determined with the addition of the Lloyd’s mirror effect by applying the modified N_PL_ to L_P_. L_RN_ and L_S_ were expressed in 1 Hz bins to provide enough frequency resolution to allow the blade lines, the frequency at which blades pass the top of the rotation, to be identified. The G-Class vessels typically transit with propeller shaft speeds of 60–70 revolutions per minute (rpm) [26], allowing for a fundamental blade rate pre-retrofitting of approximately 6–7 Hz. Because of the decrease in the number of propeller blades and the increase in rpm during the Radical Retrofit, the fundamental blade rate was reduced to approximately 4.5–6 Hz. Ocean waves become the dominant source of energy below ~5 Hz [27], and the fundamental blade rate post-retrofitting was not able to be resolved in some transits. A lower limit of 8 Hz was selected in order to ensure that the fundamental blade rate was excluded from both pre- and post-retrofitted transits. Broadband levels were computed in the frequency range of 8 Hz to 4000 Hz, by dividing into low- (8–40 Hz), mid- (40–200 Hz), high- (200–1000 Hz), and very high- (1000–4000 Hz) frequency bands. These four bands distinguish changes due to noise generated [4] by the propeller (low-frequencies), ship machinery, such as diesel engines, compressors, and pumps (high- and very high-frequencies), and the combination of these noise sources (mid-frequencies). Broadband levels were computed by summing across each of the low-, mid-, high-, and very high-frequency bands in the linear domain. Broadband levels in relation to CPA were investigated to determine if propagation loss models were over or underestimating values with distance from the HARP.

First-order polynomials were fitted to each of the sound levels in the low-, mid-, high-, and very high-frequency bands as a function of Speed Through Water using linear least-squares regression. First-order polynomials were fitted to the pre-retrofit distributions to establish baselines for L_P_, L_RN_, and L_S_. Differences between the post-retrofit sound levels and the pre-retrofit baseline levels were computed in the low-, mid-, high-, and very high-frequency bands.

### Statistical analysis

Since the G-Class vessels are sister ships with identical measurements, the twelve vessels were pooled for statistical analysis, and each transit was treated as an independent observation. An analysis of covariance was used to test for significant differences and effect size (generalized eta squared η_g_^2^), in the three reported sound levels metrics (L_P_, L_RN_, L_S_) for low-, mid-, high-, and very high-frequency bands between pre- and post-retrofit groups controlling for STW. Significant interactions between retrofit and STW were investigated to identify the influence of retrofit on sound levels for various STW.

Estimated marginal means (EMMs) controlling for STW, were computed for radiated noise and monopole source levels in 1 Hz bins and low-, mid-, high-, and very high-frequency bands. The differences between the EMMs pre- and post- retrofitting were calculated.

## Results

Opportunistic recordings of G-Class Maersk vessels were obtained for 177 transits over ten years of data collection in the SBC. Of the 177 transits, 66 transits were excluded because of the presence of singing whales, acoustic interference from other vessels, and hydrophone cable strumming, leaving 111 transits for the analysis. Acoustic recordings from all twelve of the G-Class Maersk vessels were obtained. Transits that occurred pre-retrofit and post-retrofit made up 48.7% and 51.4% of the dataset, respectively.

**Fig 4:**
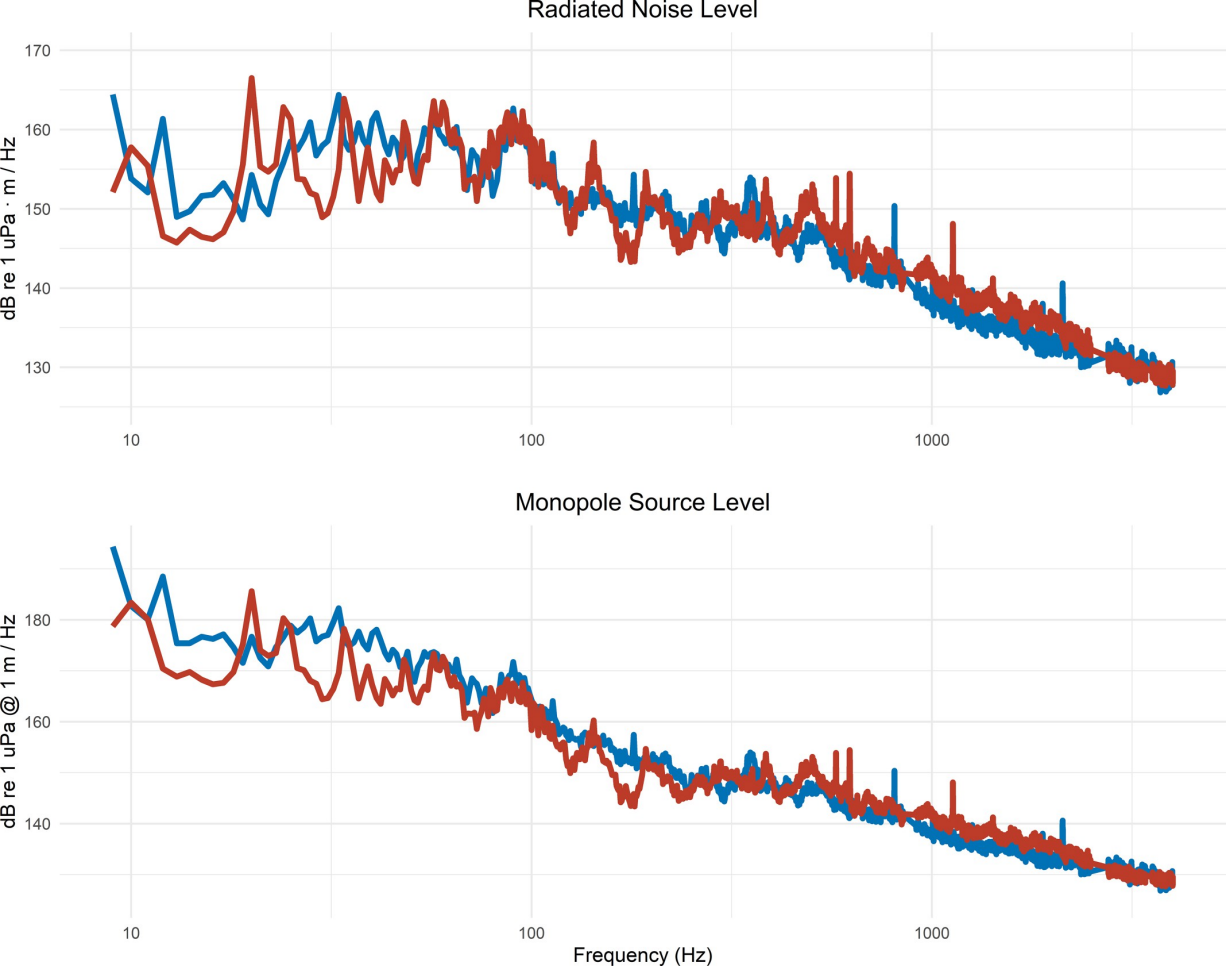
Gerda Maersk noise levels pre- and post-retrofit. Radiated noise level (top) and monopole source level (bottom) from two transits of Gerda Maersk pre-retrofit (blue) and post-retrofit (red). The pre-and post-retrofit speed through water was 6.0 and 6.1 m/s, respectively. The pre- and post-retrofit draft was 11.2 and 12.2 m, respectively.

Some parameters of the vessels changed during the Radical Retrofit. The vessel draft increased by 1.5 m on average between pre- and post-retrofit (Fig 3) owing to the expanded container capacity. Draft measurements pre-retrofit ranged from 9.6 to 13.6 m (average of 10.9 ± 0.8 m) and post-retrofit ranged from 10.5 to 15.4 m (average of 12.4 ± 1.1 m). The secondary harmonic was reduced post-retrofit from 12 Hz to 10 Hz due to the reduction in the number of propeller blades from 6 to 4 during the Radical Retrofit (Fig 4) along with a corresponding 10% increase in propeller rpm. The range (d_CPA_) of the vessel closest point of approach to the seafloor sensor decreased between pre- and post-retrofit, 4,059 ± 578 m and 3,527 ± 561 m, respectively. Spectra from two transits of the vessel Gerda Maersk at similar speed and draft pre- and post-retrofit are shown in Fig 4.

### Broadband levels

**Fig 5:**
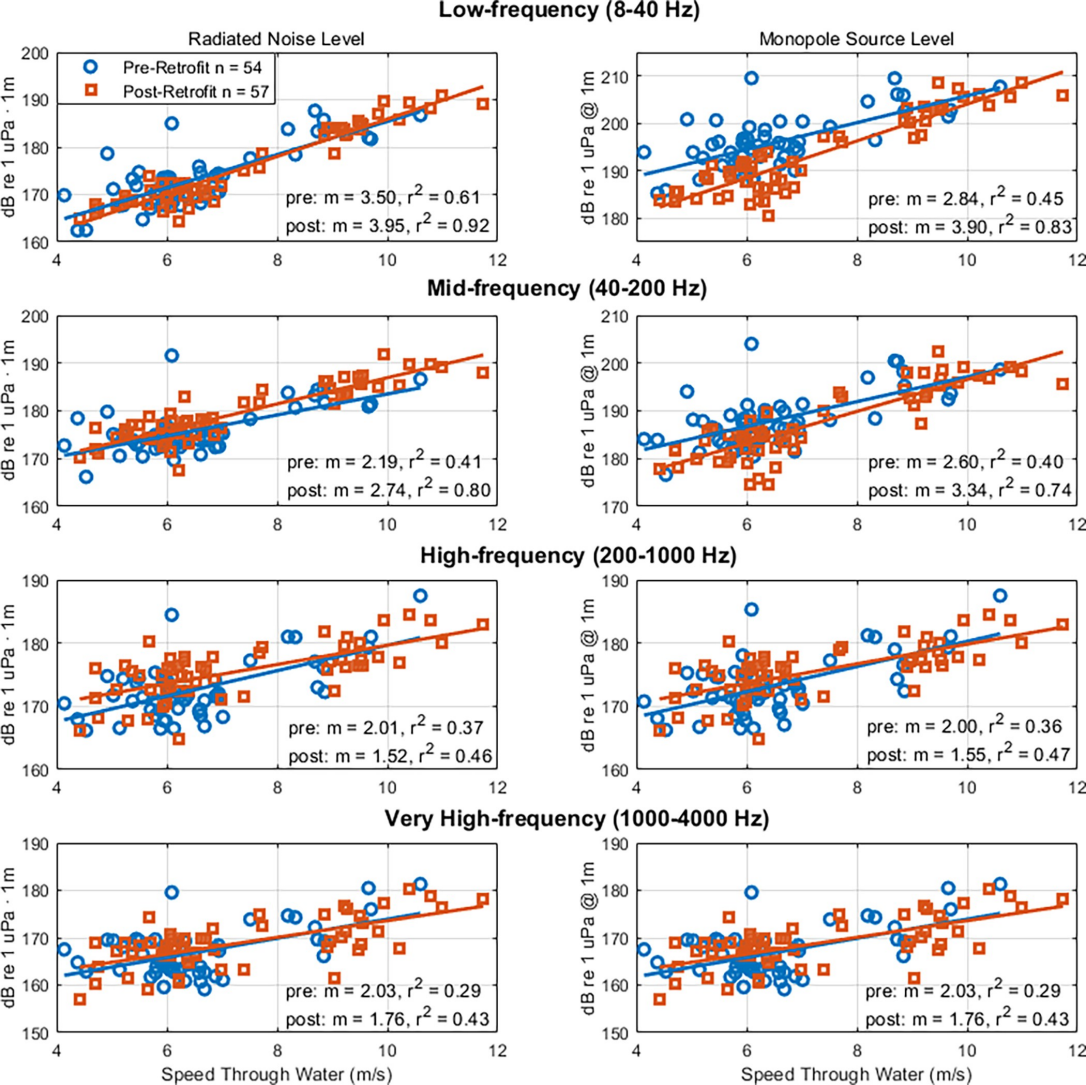
G-Class Maersk vessel noise levels in relation to speed through water. Broadband radiated noise level (left) and monopole source level (right) in relation to Speed Through Water for 111 G-Class Maersk transits pre-retrofit (blue circles) and post-retrofit (red squares) for low-, mid-, high-, and very high-frequency bands.

**Fig 6:**
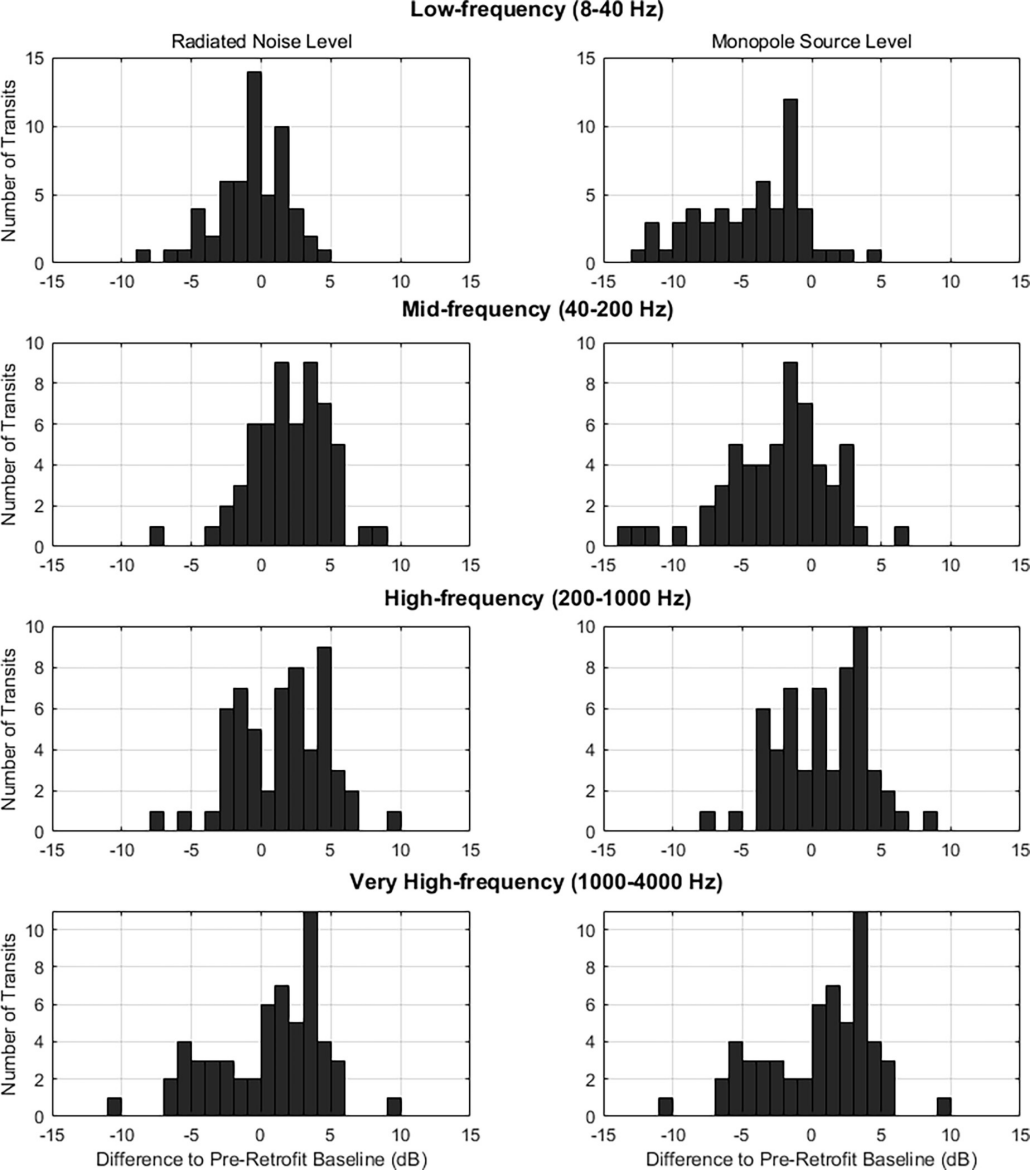
Differences in dB pre- and post- retrofit. Differences in dB between the pre- and post-retrofit radiated noise level (left), and monopole source level (right) for low-, mid-, high-, and very high-frequency bands.

At low-frequencies (8–40 Hz) the radiated noise levels pre- and post-retrofit were found to decrease by 1 dB (Figs 5, 6). In contrast, the monopole source levels were 5.2 dB lower post-retrofit (Table 2). The discrepancy between radiated noise levels (small decrease) and monopole source levels (large decrease) is owed to the increased vessel draft. An increase in draft reduces the Lloyd’s mirror effect on the radiated noise, creating higher radiated noise levels post-retrofit, whereas correcting for the Lloyd’s mirror effect resulted in lower monopole source levels post-retrofit.

**Table 2:** Radiated noise levels and monopole source levels pre- and post-retrofitting.

	Low-Frequency (8–40 Hz)		Mid-Frequency (40–200 Hz)		High-Frequency (200–1000 Hz)		Very High-Frequency (1000–4000 Hz)	
Retrofit	Radiated Noise Level	Monopole Source Level	Radiated Noise Level	Monopole Source Level	Radiated Noise Level	Monopole Source Level	Radiated Noise Level	Monopole Source Level
	L_RN_	L_S_	L_RN_	L_S_	L_RN_	L_S_	L_RN_	L_S_
Pre	174.7	197.3	176.9	189.3	173.3	174.0	167.6	167.6
Post	173.7	192.1	178.5	186.3	174.9	175.0	168.1	168.1
Δ	-1.0	-5.2	+1.6	-3.0	+1.6	+1	+0.5	+0.5
p	0.12	8.49e-10*	0.01*	3.03e-4*	0.02*	0.14	0.51	0.51
η_g_^2^	0.02	0.31	0.06	.12	0.05	0.02	0.00	0.00

Estimated marginal means (EMMs) of radiated noise level (L_RN,_ dB re 1uPa **⋅** 1m) and monopole source level (L_S,_ dB re 1uPa **⋅** 1m) in the low-, mid-, high-, and very high frequency bands pre- and post-retrofitting. The difference (Δ) between the levels is calculated by subtracting the pre-retrofit levels from the post-retrofit levels. P-values derived from the analysis of covariance between pre- and post-retrofit groups for each sound level are documented and labeled with an asterisk if p<0.05. The generalized eta squared (η_g_^2^) shows the variation explained by the retrofit covariate.

At mid-frequencies (40–200 Hz) the radiated noise levels post-retrofit increased by 1.6 dB, while the monopole source levels decreased by 3.0 dB. At high-frequencies (200–1000 Hz) and very high-frequencies (1000–4000 Hz) the radiated noise levels post-retrofit increased over those pre-retrofit (1.6 dB and 0.5 dB, respectively); whereas, the monopole source levels increased by 1 dB and 0.5 dB, respectively (Table 2), again subject to the changes in vessel draft.

The broadband radiated noise levels decreased in relation to CPA with varying slopes. The broadband monopole source levels in the low- and mid-frequencies bands did not change in relation to CPA (m = 0.001 and m = 0.000, respectively). Because of the modification of the Lloyd’s mirror model switching to N_ss_ in the higher frequencies, the high- and very high-frequency broadband monopole source levels decreased in relation to CPA.

Pre-retrofit baselines are subtracted from post-retrofit levels to provide the difference, such that a positive value is an increase and a negative value is a reduction (Fig 6). From the perspective of the vessel monopole source level, the retrofit resulted in a decreased level at low- and mid-frequencies; whereas, at high- and very high-frequencies the pre- and post-retrofit monopole source levels are comparable, suggesting that the retrofit had different effects on different noise generating mechanisms.

### Statistical analysis

**Fig 7:**
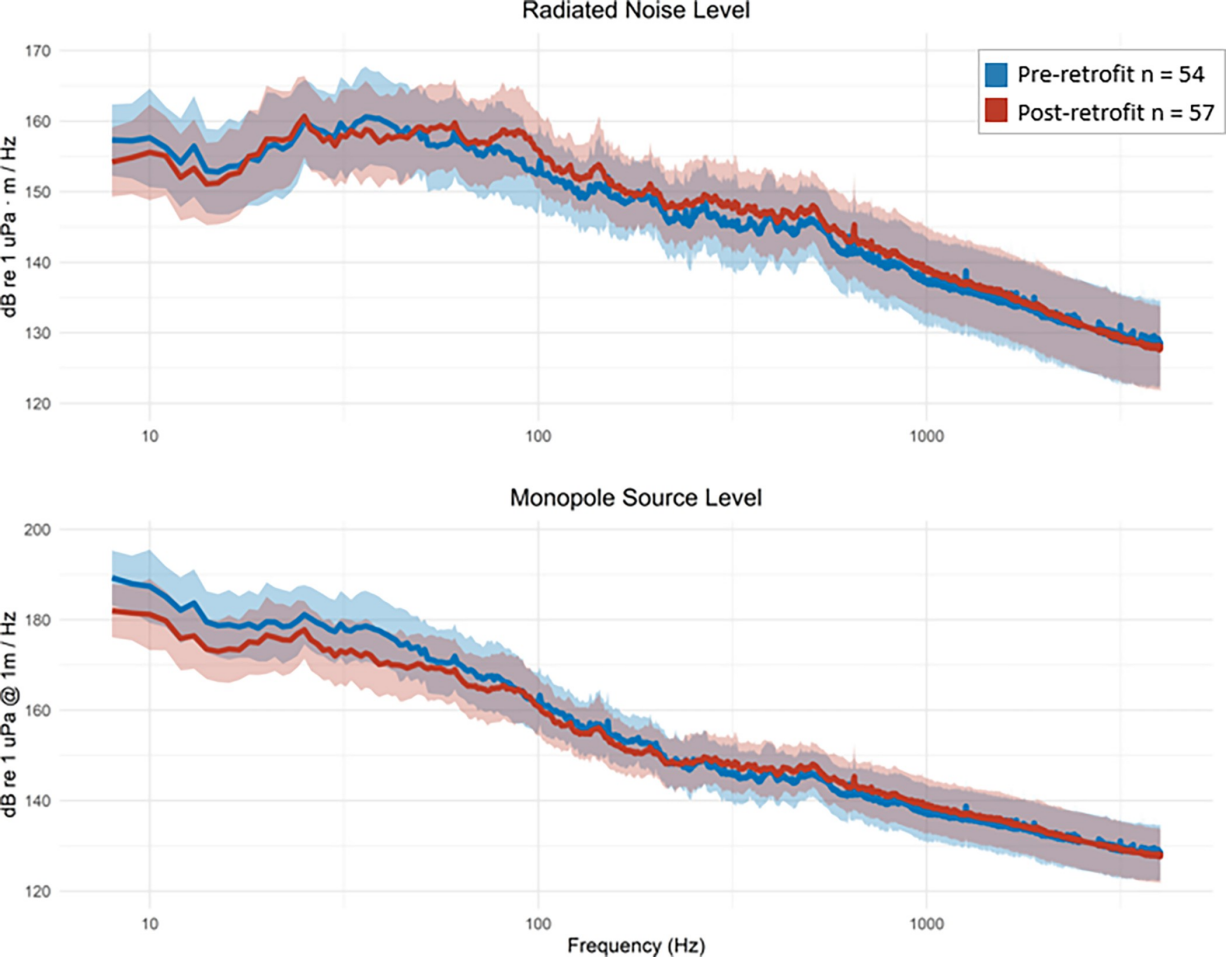
Estimated marginal means for noise levels pre- and post-retrofitting. Estimated marginal means (EMMs) ± standard deviation of radiated noise level (L_RN_) and monopole source level (L_S_) pre- and post-retrofitting.

**Fig 8:**
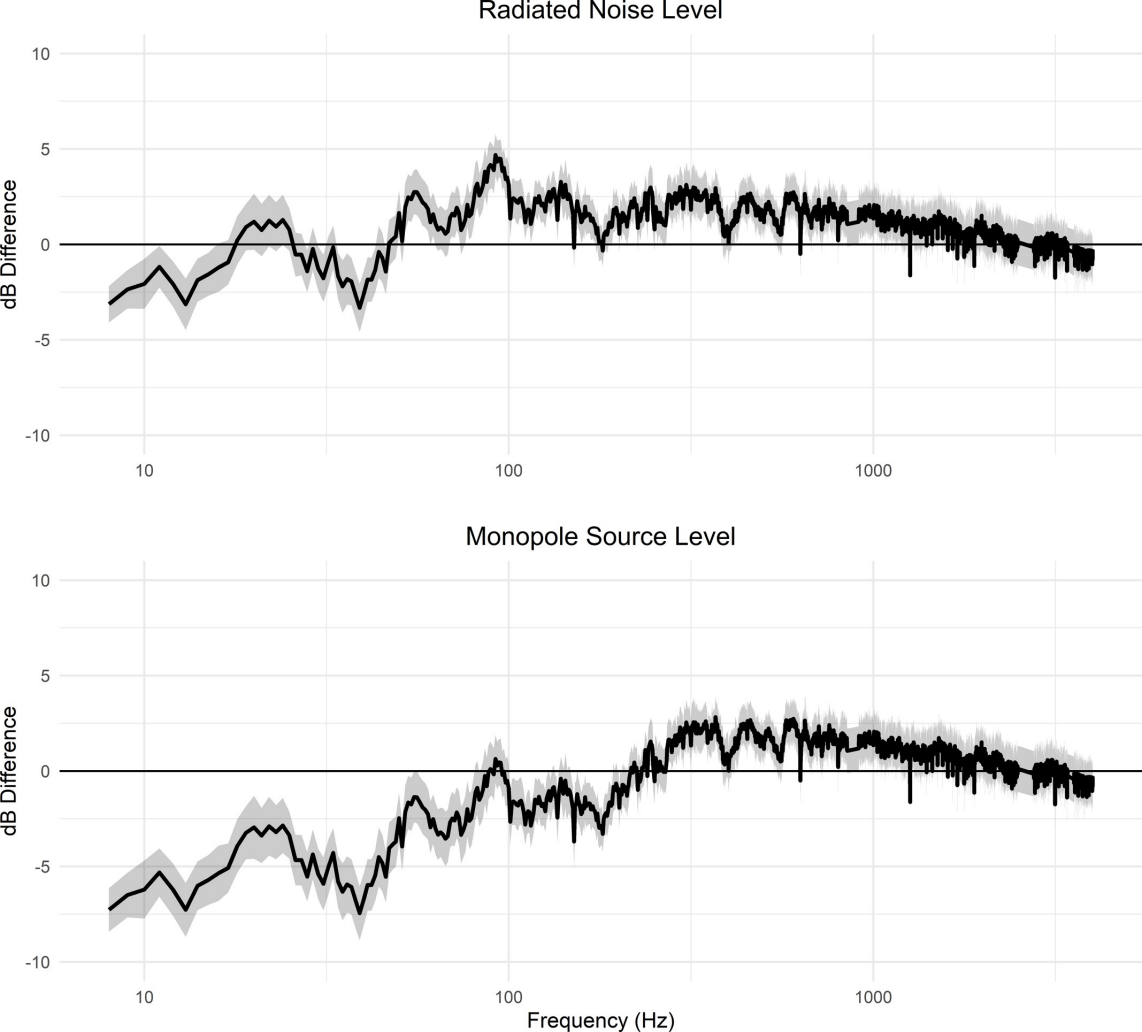
Difference in estimated marginal means for noise levels pre- and post-retrofitting. Difference +/- standard error of estimated marginal means pre- and post-retrofitting for radiated noise levels and monopole source levels.

**Fig 9:**
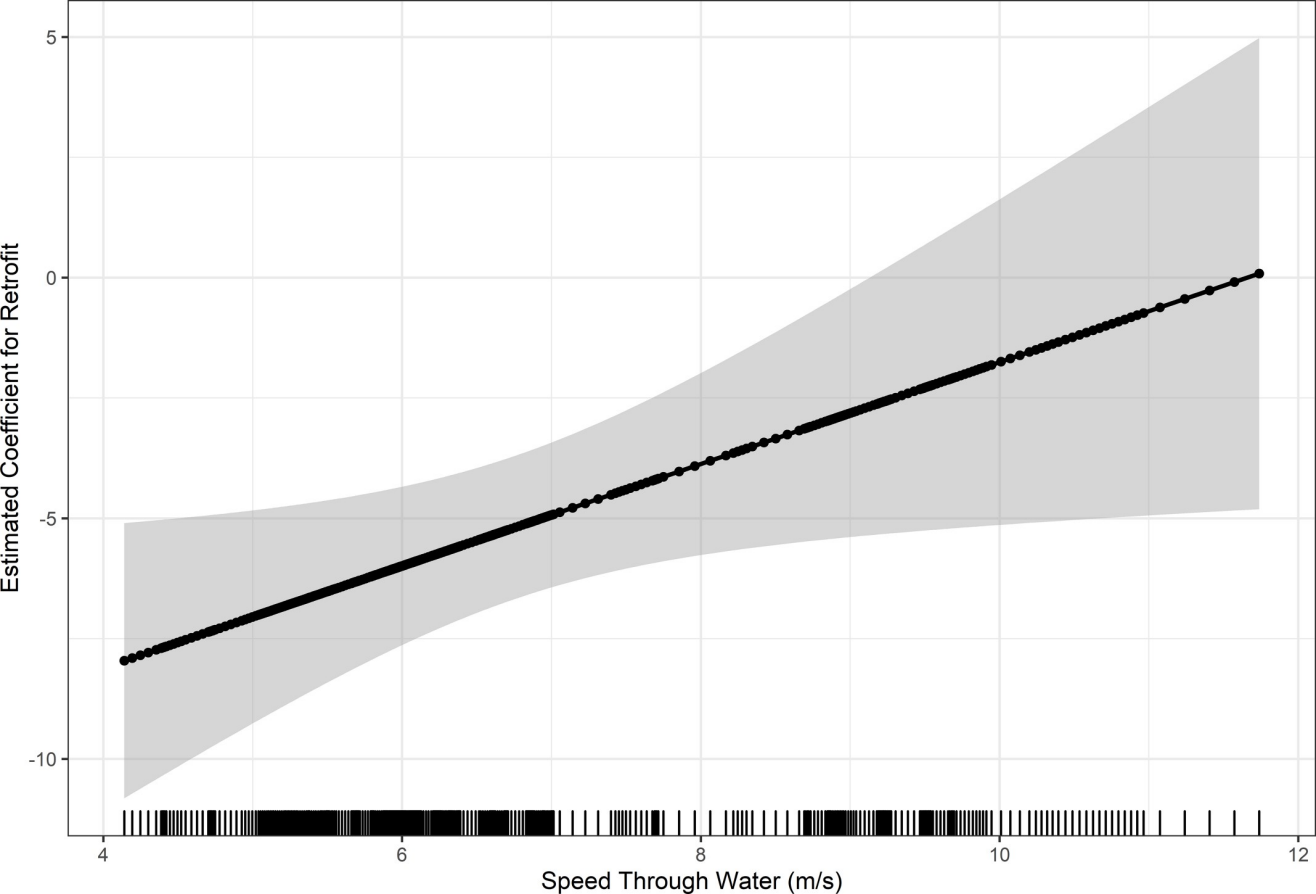
Investigation into monopole source level interaction with speed through water. Noise reduction of retrofit (dB) with Speed Through Water for low-frequency (8–40 Hz) monopole source level. Estimated coefficients and 95% confidence intervals are displayed.

When controlling for STW, the difference in the EMMs varied between pre- and post-retrofitting for radiated noise levels and monopole source level. The highest reduction was 7.5 dB at 39 Hz in the monopole source level. The highest increase was in the radiated noise level with 4.7 dB at 92 Hz (Figs 7 & 8). Retrofit had a significant effect on monopole source level in the low- and mid-frequency bands and radiated noise level in the mid- and high-frequency bands (Table 2). The explained variance (η_g_^2^) of the retrofit was the highest for the low-frequency monopole source level, with a value of 30%. However, for the monopole source level in the low-frequency band, the interaction between retrofit and STW was significant (p = 0.04). The interaction between retrofit and STW was investigated further to determine the contribution of the retrofit to sound levels for various STWs (Fig 9). The effect of retrofitting on the low-frequency monopole source level ranges from -7 dB at 4 m/s (7.8 knots) to 0 dB at 12 m/s (23.3 knots), suggesting that the primary impact of the retrofit was to reduce source levels when the vessel is operated at low speed. The 95% confidence intervals of the coefficients are wider for the high speeds, mostly likely because there were fewer transits at speeds > 10 m/s contributing to the model.

## Discussion

Retrofitting efforts undertaken by Maersk were investigated for the potential of vessel noise reduction. A long-term passive acoustic dataset spanning over ten years in the SBC was used to estimate radiated noise levels and monopole source levels for more than 100 transits of Maersk G-Class vessels pre- and post-retrofit. With multiple changes to the design of the sister ships undertaken during the Radical Retrofit, there were several possible sources for the changes to sound levels highlighted in this study. An increased demand in cargo led to an increase in maximum draft during the Radical Retrofit, which led to increased cargo capacity. From one perspective, the increased cargo capacity will lead to fewer trips and thereby reduce ocean noise, however, this will only be true if global demand stays constant. From the perspective of the radiated noise level of individual vessels, a deeper draft increases the noise output, owing to a decrease in Lloyd’s mirror effect at low-frequencies. The radiated noise levels are higher post-retrofit because they are not corrected for the depth of the acoustic source. Additionally, the CPA pre-retrofit was approximately 500 m farther from the sensor, which may have underestimated total levels by 2 dB in the high- and very high-frequency bands, making the difference between pre- and post-levels -1.5 to 0 dB (S1 Fig). The low-frequency monopole source level is reduced because it accounts for the Lloyd’s mirror effect, making it possible to better understand the contribution of components of the retrofit, other than the draft, to noise-generation by the vessel. Without the correction for source depth and image source interference, this study would not have resolved the monopole source level reduction in the low- and mid-frequency bands. Without an increase in TEU capacity post-retrofit and subsequent increases in draft, there might have been a reduction in radiated noise in addition to monopole source level; additional experiments studying this effect should be conducted. Overall, the actual sound power radiated by the ships is affected by Lloyd’s mirror, making the draft a key parameter for vessel noise measurements.

### Retrofit interaction with speed

The interaction between retrofitting and STW was significant for the low-frequency monopole source level, highlighting that the retrofit-induced quieting is speed dependent. The propeller design post-retrofitting was developed for higher efficiency at slower speeds, which may be the cause of the reduction of source level due to retrofitting. Additionally, the 95% confidence intervals of the interaction were wider at high speeds because of the low number of transits obtained traveling at speeds faster than 10 m/s (19.4 knots). There were five post-retrofit transits faster than 10 m/s, but only one pre-retrofit transit faster than 10 m/s. As more shipping lines retrofit their vessels, this interaction should be investigated and verified.

### Source of noise reduction

There were multiple changes during the Radical Retrofit effort undertaken by Maersk, including changes to the bow, propellers, and engine. To disentangle the effects of those changes, this study highlighted four frequency bands. Since the monopole source level noise reduction is limited to the low- and mid-frequency bands and is greatest at low speed, the noise improvement is probably related to reduction in propeller cavitation at low speed. Based on a 9 m diameter propeller turning at 60 rpm, the blade tip speed pre-retrofit would be 28.3 m/s. This is less than the post-retrofit speed of 32.1 m/s obtained from a 9.3 m diameter propeller turning at 66 rpm. Higher propeller tip speed should result in increased cavitation, so other factors must have played a decisive role in reducing the net cavitation. The addition of propeller boss cap fins most likely minimized hub vortex cavitation and subsequent vibrations in the rudder and shaft. The reduction in propeller blades and area may have reduced the number of cavitation inception points. Modifications to the bulbous bow may be another factor since this could reduce vessel resistance allowing the propeller to operate at a lower rpm, reducing propeller cavitation. Further studies and modeling would be required to estimate the degree to which each of these factors contributed to the monopole source level reduction.

## Conclusion

A reduction of monopole source level in the low-frequency band following a Radical Retrofit effort undertaken by Maersk is identified in this study. Although there were many alterations to the ship design during retrofitting, the reduction in the low-frequency band suggests that noise reduction was due to the changes in the propeller and bow design. The interaction between retrofit and speed in this study was significant, highlighting that the effect of retrofitting on monopole source level was greatest at slower speeds. As more shipping lines are implementing design changes for lower carbon shipping, source level reductions due to modifications should be investigated to reveal which alterations lead to the greatest reductions. Additionally, the interaction between retrofitting and speed found in this study should be tested in future analyses.

Ship design specifications and technologies for the reduction of noise generated by commercial ships have been identified by the International Maritime Organization with the intention to reduce ship noise on an international level. However, the specific goals of noise reduction need to be further developed. For instance, this study has demonstrated that ~2,000 more TEU can be moved with only a 0–2 dB increase in radiated noise level per transit, resulting in a reduction in noise per TEU, and a potential 20% reduction in transits. This study also demonstrated that the retrofit design efforts could reduce radiated noise levels per transit if not for the requirement to increase TEU capacity. Therefore, the IMO, IWC, and additional parties considering noise reduction will need to refine the goal of noise reduction, whether that be reducing transits, reducing noise per TEU, or reducing noise per transit. Future studies should focus on testing the reductions found in this study with larger sample sizes, different ship types, and different design approaches to identify the most efficient methods for reducing underwater noise on an international level.

## Supporting information

S1 Fig Supplemental analysis of broadband noise levels. Radiated noise levels and monopole source levels in relation to Closest Point of Approach (CPA) in low-, mid-, high-, and very high-frequency bands. Signal to noise ratio for each transit in relation to CPA.
(TIFF)

S1 Data MAERSK retrofit. (CSV)
