# Supplementary figures and images for: Retrofit-induced changes in the radiated noise and monopole source levels of container ships
Source: PLoS One. 2023 Mar 16;18(3):e0282677. doi: 10.1371/journal.pone.0282677 (PMC10019734; doi:10.1371/journal.pone.0282677)

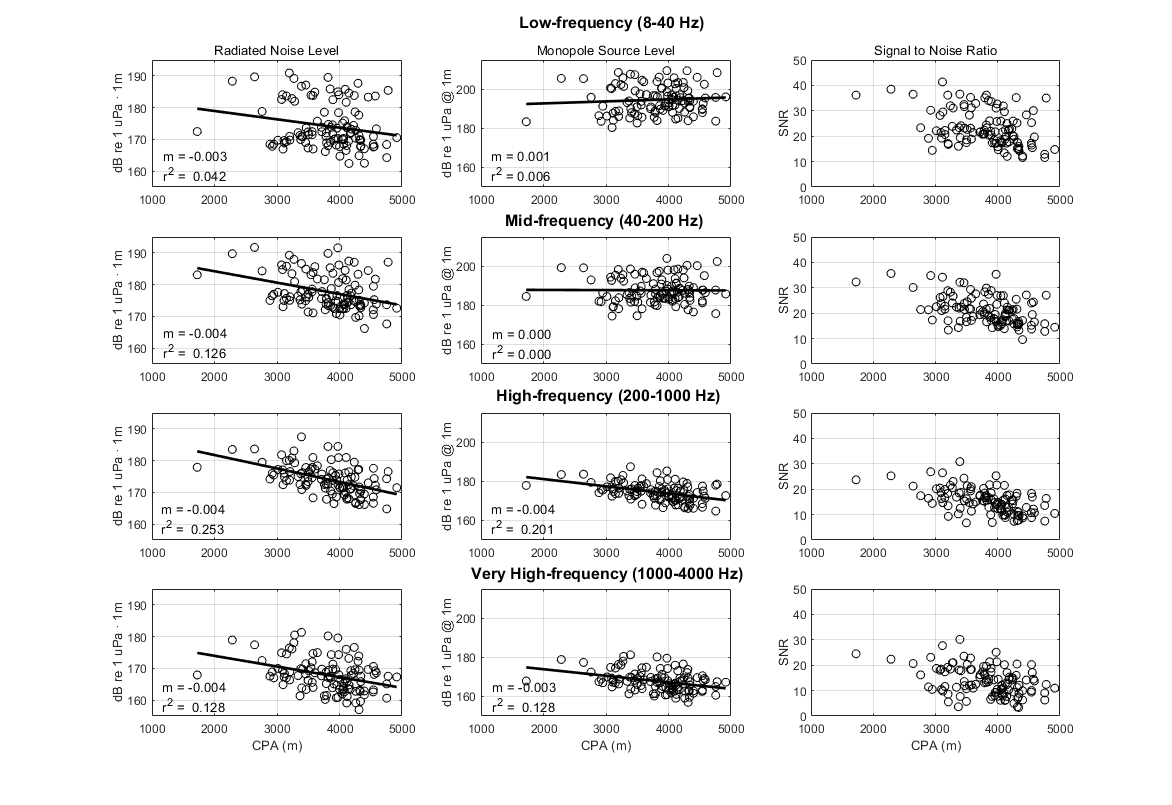

Supplement: S1 Fig — Radiated noise levels and monopole source levels in relation to Closest Point of Approach (CPA) in low-, mid-, high-, and very high-frequency bands. Signal to noise ratio for each transit in relation to CPA. (TIFF) [file pone.0282677.s001.tiff]
